# Curcumin Alleviates Doxorubicin-Induced Cardiotoxicity by Modulating Apelin Expression

**DOI:** 10.3390/biom15101416

**Published:** 2025-10-05

**Authors:** Baris Akca, Olcay Murat Disli, Nevzat Erdil, Yilmaz Cigremis, Hasan Ozen, Merve Durhan, Selahattin Tunc, Onural Ozhan, Zeynep Ulutas, Feray Akgul Erdil

**Affiliations:** 1 Department of Cardiovascular Surgery, Faculty of Medicine, Inonu University, 44280 Malatya, Türkiye; olcaydisli@hotmail.com (O.M.D.); nevzat.erdil@inonu.edu.tr (N.E.); 2 Department of Medical Genetics, Faculty of Medicine, Inonu University, 44280 Malatya, Türkiye; yilmaz.cigremis@inonu.edu.tr (Y.C.); mervedurhan@windowslive.com (M.D.); selahattintunc23@gmail.com (S.T.); 3 Department of Pathology, Faculty of Veterinary Medicine, Balikesir University, 10145 Balikesir, Türkiye; hasanozen@hotmail.com; 4 Department of Pharmacology, Faculty of Medicine, Inonu University, 44280 Malatya, Türkiye; onural.ozhan@inonu.edu.tr; 5 Department of Cardiology, Faculty of Medicine, Inonu University, 44280 Malatya, Türkiye; zeynep.ulutas@inonu.edu.tr; 6 Department of Anesthesiology, Faculty of Medicine, Inonu University, 44280 Malatya, Türkiye; feray.erdil@inonu.edu.tr

**Keywords:** cardioprotection, oxidative stress, anthracycline toxicity, apelinergic signaling, heart failure

## Abstract

**Background:** Doxorubicin (Dox)-induced cardiotoxicity is the most important side effect of the drug and significantly limits its use in susceptible patients. Therefore, preventive measures are required to alleviate the Dox-induced cardiac failure. In this study, curcumin, a strong antioxidant agent, was investigated for its potential protective effect on dox-induced cardiotoxicity with its effect on Apelin expression as a mediator of cardiac function. **Methods:** Wistar albino rats were equally divided into four groups as Control, DOX, CUR, and CUR+DOX. Dox was administered a single dose of 20 mg/kg bw intraperitoneally while 100 mg/kg bw curcumin was given orally for 14 days before the Dox use. **Results:** DOX group showed a prolonged QT interval on an electrocardiogram and elevated cardiac troponin levels. In biochemical analyses, decreased Superoxide Dismutase activity and increased Malondialdehyde level and Catalase activity were detected in DOX group. Gene expression of *Apelin* decreased significantly while *NF-κB* increased in DOX group. Degenerative changes in histopathology, and increased iNOS and nitrotyrosine immunoreactivity were detected in DOX group. However, no significant changes were observed at reduced Glutathione, TNF-, and IL-1β levels. Curcumin use in Dox-given rats altered most of the disturbed parameters investigated in this study, indicating an alleviating effect on Dox-induced cardiotoxicity. Serum and heart Apelin levels and mRNA expression in heart tissue were detected to significantly increase in CUR+DOX group as compared to DOX group. Furthermore, *NF-κB* mRNA expression was significantly decreased in heart tissue of CUR+DOX group compared with the DOX group. **Conclusions:** The results suggest that Apelin acts as an important mediator in Dox cardiotoxicity and may be used as a target for treatment of certain cardiomyopathies. By regulating Apelin expression, curcumin may serve as a potential adjunct in cardioprotective approaches.

## 1. Introduction

Doxorubicin (Dox) is a commonly used anthracycline antibiotic in the treatment of many types of tumors. Despite its clinical significance and wide use, its cardiotoxic effects are one of the most important factors limiting the treatment potential. Therefore, substances alleviating the cardiotoxic effect of the agent bear important clinical benefits.

A detailed definition of cancer therapy-related cardiac dysfunction was established in 2022, and clinically can be identified by the onset of heart failure symptoms such as dyspnea, fatigue, chest pain, and shortness of breath while paraclinically it is identified by electrocardiographic changes such as QT prolongation and arrhythmias [1]. Dox toxicity is associated with production of reactive oxygen species (ROS), mitochondrial impairment, inflammation, and apoptotic processes [2]. Dox can cause tissue damage by affecting natural antioxidant defense mechanisms such as Copper Zinc Superoxide Dismutase (CuZn-SOD), Catalase (CAT), and reduced Glutathione (GSH), particularly in the heart muscle [3]. Biochemical reflections of cardiotoxicity include increased Malondialdehyde (MDA) associated with lipid peroxidation, changes in antioxidant defense mechanisms, and elevated cardiac injury markers such as cardiac troponin (cTn) [4]. It has been reported that not only oxidative stress, but also inflammatory cytokines play a role in the pathogenesis of Dox-induced cardiotoxicity. Dox induces a prominent increase in pro-inflammatory cytokines. Increases in the production of tumor necrosis factor (TNF)-α, interleukin (IL)-1β, and IL-6 via nuclear factor-κB (NF-κB) activation were described in Dox toxicity [5,6,7].

Apelin is an endogenous peptide synthesized from the *APLN* gene and can be expressed in many organs such as the heart, lungs, kidneys, and liver [8]. Apelin, which appears as one of the most powerful stimulants of cardiac contraction in adults, can also act as a mediator of cardiovascular control, and is involved in the regulation of blood pressure and blood flow [9]. In clinical conditions such as heart failure and atherosclerosis, the gene expression and circulating levels of Apelin may decrease or stay unchanged [10]. It has been suggested that Apelin may exhibit anti-inflammatory effects by suppressing the excessive production of pro-inflammatory cytokines such as TNF-α and interleukins [11].

Curcumin is a potent polyphenolic compound derived from *Curcuma longa*. Thanks to its strong antioxidant, anti-inflammatory, and anti-apoptotic properties, it was suggested to show cardioprotective effects [4,12]. Curcumin was shown to decrease cTn level in Dox-induced cardiotoxicity, indicating a protective effect on heart tissue [13]. It has also been suggested that curcumin may have multiple cardioprotective effects due to its antioxidant, anti-inflammatory, and anti-apoptotic effects in Dox-induced cardiotoxicity [14].

In this study, the mechanisms of the possible alleviating effect of curcumin in the prevention of cardiotoxicity induced by Dox were evaluated by studying *Apelin* and *NF-κB* gene expressions, changes in cTn level, electrocardiography (ECG) alterations, oxidative stress parameters, tissue damage, and histopathologic changes.

## 2. Materials and Methods

### 2.1. Experimental Design

The study protocol was approved by the permission of Institutional Animal Ethics Committee of Inonu University (Approval code: 2023/11-7; Approval date: 9 November 2023). A total of 40 Wistar albino rats were used in the experiment. The rats were kept in a temperature (21 °C) and humidity (55–60%) controlled room with 12 h light and dark cycles. Throughout the study, the animals were fed ad libitum with standard dry rat diet and tap water. The rats were equally divided into four groups, each containing 10 animals, and named as follows: Control (C), Curcumin (CUR), Doxorubucin (DOX), Curcumin+Doxorubucin (CUR+DOX). Saline (ip, vehicle for DOX) and corn oil (orally, vehicle for curcumin) were administered to the control (C) group for 14 days. The CUR group was given oral curcumin (100 mg/kg bw) for 14 days. In the Doxorubucin group (Adriamycin-Deva Holding A.Ş., İstanbul, Türkiye), a single dose of 20 mg/kg bw was given ip on the last day of the 14-day-period [15]. In the CUR+DOX group, curcumin at 100 mg/kg body weight was given ip for 14 days, and DOX at 20 mg/kg bw was applied as a single dose on the last day of the 14-day-period. After the 14th day of the experiment, the rats were kept for 48 h to allow the development of cardiotoxicity and monitored daily.

### 2.2. Blood Pressure Measurement

On the final day of the experiment, following anesthesia induction with urethane (1.2 g/kg, ip, ethyl carbamate, CAS: 51-79-6), a midline cervical incision was made, and the right common carotid artery was carefully isolated and cannulated using a PE-50 catheter filled with heparinized saline (50 IU/mL). The catheter was connected to a pressure transducer coupled to the Biopac MP100 data acquisition system (BIOPAC Systems Inc., Goleta, CA, USA) to enable continuous invasive monitoring of systolic blood pressure (SBP), diastolic blood pressure (DBP), and mean blood pressure (MBP). Blood pressure and ECG signals were recorded simultaneously while the animal was in lateral recumbency. The average of three stable 10 s segments was used for analysis.

### 2.3. ECG Measurement

Rats were placed in lateral recumbency. A standard lead II ECG derivation was obtained using needle electrodes inserted subcutaneously into the right forelimb, left forelimb, and left hind limb. ECG signals were recorded using a Biopac MP100 data acquisition system (BIOPAC Systems Inc., Goleta, CA, USA), with a paper speed of 50 mm/s and a sensitivity of 10 mm/mV. Each ECG was recorded for at least 1 min to assess rhythm stability and to calculate heart rate (HR), PR interval, QRS duration, and QT interval. Conduction delay was accepted as the prolongation of PR or QRS intervals consistent with atrioventricular block or intraventricular conduction slowing. One-minute tracings were considered adequate to evaluate both baseline rhythm and waveform morphology, as rats typically have high resting heart rates and stable rhythms under urethane anesthesia.

### 2.4. Sample Collection

The rats were euthanized by exsanguination under urethane anesthesia. Before the animals were euthanized, blood samples were collected from the inferior vena cava. Serum samples were obtained by centrifugation, and the samples were saved at −80 °C for biochemical analyses. The hearts were collected and then cut into two pieces in the longitudinal direction to obtain both the atrial and ventral cardia. One half was chopped into small pieces and placed in an RNA saving solution and then saved at −80 °C for molecular analyses or freshly used for biochemical analyses. The remaining half was preserved for histological and immunohistochemical analysis in 10% neutral buffered formalin.

### 2.5. Biochemical Analyses in Heart Tissue

MDA level in the heart homogenates was detected according to the method described by Mihara and Uchiyama [16] and the results were presented as nanomoles of MDA per gram of wet tissue (nmol/gwt). GSH levels were measured using the method mentioned by Ellman [17]. GSH was reacted with 5,5-dithiobis-2-nitrobenzoic acid and the level was detected spectrophotometrically at a wavelength of 410 nm. The results were presented as nmol/gwt. CuZn-SOD activity was measured using the method described by Sun et al. [18]. The results are presented as U/gr protein. CAT was determined according to the method described by Lück [19]. CAT activity in the heart tissue samples was measured at 240 nm and expressed as K/g protein.

### 2.6. Quantification of Ctn, Apelin, Tnf, Il-1β

Troponin-I (Ref No: QS0713Ra, Sunlong Biotech Co., Ltd., Hangzhou, China), Apelin (REF No: QS0993Ra, Sunlong Biotech), interleukin-1β (IL-1β) (Ref No: QS0402Ra, Sunlong Biotech) and tumor necrosis factor-α (TNF-α) (Ref No: QS0722Ra) were analyzed with ELISA assay from the serum of rats in accordance with the protocol of the manufacturer (Lot No: 20240925 Sunlong Biotech).

### 2.7. RT-qPCR for mRNA Expression of Apelin and Nf-Κb

**Figure 1:**
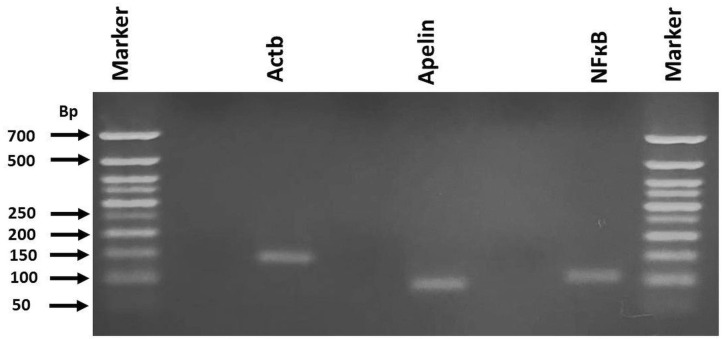
The PCR product size of *Actb*, *Apelin,* and *NF-кB* genes from the groups. RNA extractions from the heart tissues were performed and PCR amplification of the target genes were performed using the primer pairs designed for *Actb*, *Apelin,* and *NF-кB*. PCR products were run on 1.5% agarose gels. 50 bp DNA ladder was used as comparison.

Total RNA was extracted with an RNA Tissue Kit (RNeasy Fibrous Tissue Mini Kit, AllPrep RNA FFPE Kit, Lot: 178027838, Ref No: 74704, Qiagen, Venlo, The Netherlands). cDNA synthesis was performed by the QuantiTect Reverse Transcription Kit (Lot: 178026669, Ref No: 205311, Qiagen). Real-time PCR was performed in a Light Cycler Instrument (Roche Applied Science, Penzberg, Germany) using QuantiNova SYBR Green PCR Master Mix Kit (Lot: 178023497, Ref No: 208054, Qiagen) and Real-Time Ready Assay *β-actin (Actb)* (Lot:1110906123191, Qiagen), *Apelin* (Lot:11109061231101, Qiagen), *NF-κB* (Lot:1110906123161, Qiagen) with the primer pairs listed in Table 1. Relative mRNA expressions were calculated using the *Actb* gene as the housekeeping gene using the 2^−ΔΔ^Ct method [20]. PCR products were run on DNA agarose gel and the expected PCR products of 139 bp, 93 bp, and 103 bp were examined for the identification of the *Actb*, *Apelin,* and *NF-κB* genes, respectively (Figure 1).

### 2.8. Histopathological Analysis

Heart tissue samples were fixed in 10% buffered formalin solution and then embedded in paraffin. Five-um-thick sections were taken from the paraffin blocks and placed on polylysine-coated slides. The tissue sections were routinely stained with hematoxylin and eosin (HE) and evaluated under a light microscope for the presence of pathological changes.

### 2.9. Immunohistochemical Analysis

The expression of inducible nitric oxide synthase (iNOS) and nitrotyrosine in heart tissue samples was investigated using the avidin–biotin peroxidase technique. Briefly, the heart sections obtained from the paraffin blocks were deparaffinized and treated with H_2_O_2_ to block endogenous peroxidase activity. Non-immune goat serum was used to block non-specific antibody binding. Anti-iNOS (Cat No: PA5-16855, Invitrogen, Waltham, MA, USA) and anti-nitrotyrosine antibodies (Cat No: AB5411, Millipore, Burlington, MA, USA) at 1:300 dilutions were applied onto the sections for 1 h incubation. Then biotinylated secondary antibody followed by a streptavidin–biotin immunoperoxidase complex was applied for 30 min each. Peroxidase activity was revealed by a 3,3-diaminobenzidine/H_2_O_2_ solution. Finally, the sections were stained with hematoxylin for background staining and observed under a light microscope. Evaluation of immunohistochemical staining was performed semi-quantitatively according to the following criteria. No staining; 0, poor staining; 1, medium staining; 2, severe staining; 3.

### 2.10. Statistical Analysis

Power analysis revealed minimum of 10 subjects per group to detect a statistically significant difference, with a predicted effect size of 0.05 Type I error (alpha), 0.8 test power (1-beta) and 1.04. Therefore, a total sample size of at least 40 was necessary. The Shapiro–Wilk test was employed to evaluate the conformity of the quantitative data to the normal distribution. However, the data did not demonstrate a normal distribution; consequently, they were presented with a median and interquartile range (25–75 P). The Kruskal–Wallis H test was utilized for intergroup comparison of data, and the Conover test was employed for post hoc analysis. All analyses were performed using MedCalc Version 19.7 for Windows statistical software package for biomedical research. Any *p* value of 0.05 was considered to demonstrate statistical significance.

## 3. Results

### 3.1. Electrocardiogram (ECG)

The results of the ECG are presented in Table 2. The heart rate was significantly elevated in the DOX and CUR+DOX groups as compared to the C and CUR groups (*p* < 0.05). Similarly, systolic, diastolic, and median blood pressures were also significantly higher in the DOX and CUR+DOX groups (*p* < 0.05). The PR and QRS intervals did not show any significant differences among the groups. The QT interval was significantly prolonged in the DOX group as compared to the other groups (*p* < 0.05) and curcumin use in Dox-given rats significantly shortened the QT interval to a time similar to the control group.

In the ECG measurements, no pathological changes were observed in the control and CUR groups. In the DOX group, one rat had ST depression, two rats had T wave negativity, and three rats had conduction delay. In the CUR+DOX group, one rat had ST depression, one rat had ST elevation, one rat had T wave negativity, and one rat had conduction delay. Adding curcumin to the Dox-given rats improved conduction delay by reducing it. The ST changes and T wave negativity persisted in the CUR+DOX group, while curcumin had no effect.

### 3.2. Biochemical Analyses

The results of cardiac and serum troponin, Apelin, TNF-α, and IL-1β analyses are summarized in Table 3. The level of cTn increased significantly in the DOX group as compared to the C and CUR groups while it comparably decreased in the DOX+CUR group (*p* < 0.05). Serum Apelin level significantly decreased in the DOX group as compared to the other groups (*p* < 0.05). Heart Apelin level also showed some decrease in the DOX group, but was not significant; in the DOX+CUR group, it was significantly higher as compared to the DOX group (*p* < 0.05). TNF-α and IL-1β levels both in serum and heart tissue did not show significant differences among the groups.

The results of MDA, GSH, CuZn-SOD, and CAT levels in the heart tissue samples are summarized in Table 4. The MDA level was significantly increased in the DOX group as compared to the other groups (*p* < 0.05), and curcumin addition to Dox-given rats significantly decreased the MDA level similar to that of the control group. Similar results were also observed for both CuZn-SOD and CAT levels in that CuZn-SOD activity in the DOX group was significantly decreased while CAT activity increased and the results were significantly different from the other groups (*p* < 0.05). No significant differences were observed for GSH level among all the groups in this study (*p* > 0.05).

### 3.3. Apelin and NF-κB Gene Expression

**Figure 2:**
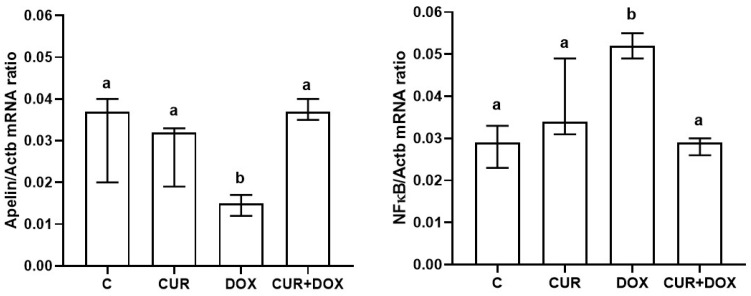
The mRNA expressions of *Apelin* and *NF-кB* in the heart tissues. mRNA expressions of *Apelin* and *NF-кB* were assessed by real-time RT-PCR. Data are summarized as median (interquartile range; 25–75%). Different letters on each bar for a given parameter are statistically significant (*p* < 0.05). C: control, CUR: curcumin, DOX: Doxorubucin.

*Apelin* and *NF-κB* gene expression levels are shown in Figure 2. *Apelin* gene expression was significantly decreased in the DOX group as compared to the other groups, and curcumin addition significantly increased the gene expression similar to that of the C group (*p* < 0.05). *NF-κB* gene expression was significantly increased in the DOX group as compared to the other groups and curcumin addition significantly decreased the gene expression (*p* < 0.05).

### 3.4. Histopathology

**Figure 3:**
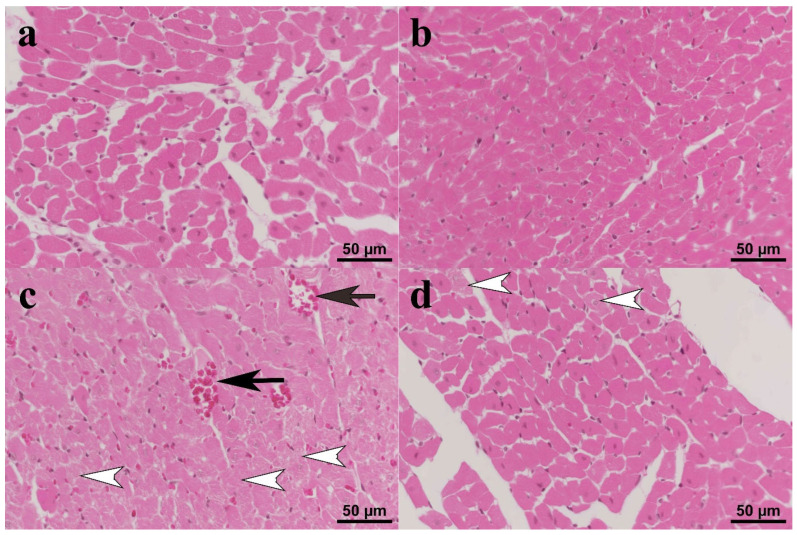
(**a**) Control: Normal histomorphology of the cardiac tissue; (**b**) CUR: No histopathological changes; (**c**) DOX: Severe degeneration characterized by vacuolation in the cytoplasm of some cardiomyocytes (white arrow heads) and hyperemia (black arrows); (**d**) CUR+DOX: Weak to moderate degeneration in some cardiomyocytes (white arrow heads). HE.

Normal histomorphology was observed in the heart samples of the C (Figure 3a) and CUR (Figure 3b) groups. Histopathological changes ranging from moderate to severe were observed in the DOX group. The myocytes in the heart tissues in this group were swollen and had lost their lamellation. Some myocytes had pyknotic nuclei, while others had completely lost their nuclei. Hyperemia was observed in all sections (Figure 3c). In the CUR+DOX group, some myocytes exhibited degenerative changes, but these changes were significantly reduced compared to the DOX group. Occasional hyperemia was observed in a small number of cases in this group, while no evidence of hyperemia was observed in the other cases (Figure 3d).

### 3.5. Immunohistochemistry

**Figure 4:**
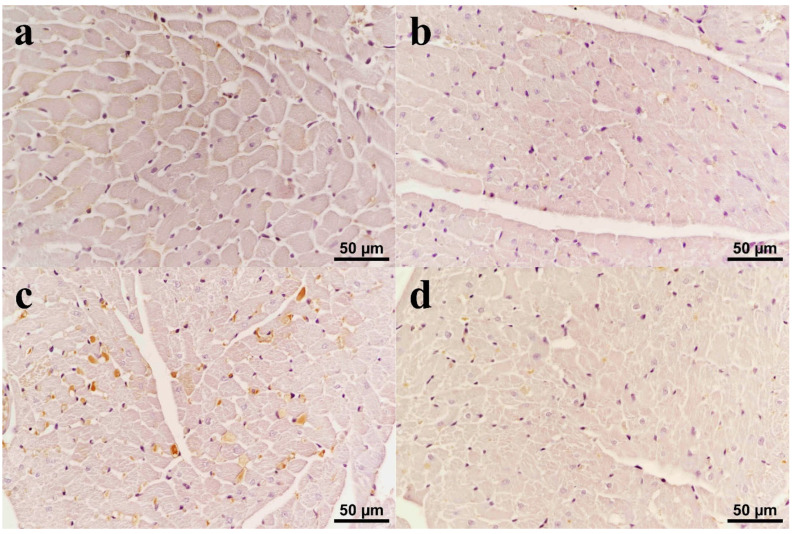
iNOS immunoreactivity in heart tissues. (**a**) Control: Weak intracytoplasmic immunoreactivity in myocytes; (**b**) CUR: Weak immunoreactivity in myocytes; (**c**) DOX: Severe intracytoplasmic iNOS immunoreactivity in many cardiomyocytes; (**d**) CUR+DOX: Weak to moderate immunoreactivity in occasional myocytes.

Immunohistochemical staining for iNOS revealed mild intracytoplasmic immunoreactivity in myocytes in heart tissues in the C (Figure 4a) and CUR (Figure 4b) groups. In the DOX group, iNOS immunoreactivity was detected in numerous degenerative myocytes, mostly at a high level (Figure 4c). Mild to moderate iNOS immunoreactivity was detected in subjects both with both Dox and curcumin (Figure 4d).

**Figure 5:**
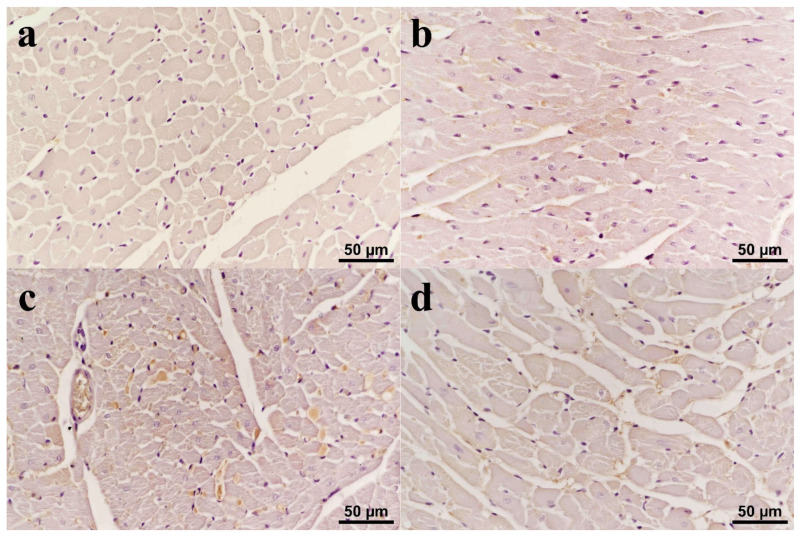
Nitrotyrosine immunoreactivity in heart tissues. (**a**) Control: Weak intracytoplasmic immunoreactivity in myocytes; (**b**) CUR: Weak immunoreactivity in myocytes; (**c**) DOX: Moderate nitrotyrosine immunoreactivity in degenerated cardiomyocytes; (**d**) CUR+DOX: Weak nitrotyrosine immunoreactivity in myocytes.

Immunohistochemical staining for nitrotyrosine revealed mild intracytoplasmic staining in myocytes in heart tissues from subjects in the C (Figure 5a) and CUR (Figure 5b) groups. In the DOX group, nitrotyrosine immunoreactivity was observed in myocytes, mostly at a moderate level (Figure 5c). When the nitrotyrosine immunoreactivity in CUR+DOX group was compared with DOX group, it was found that the staining was only slightly intense and thus significantly reduced (Figure 5d). Statistical analysis of the immunohistochemical staining for both iNOS and nitrotyrosine showed significant increases in immunoreactivities in the DOX groups compared to the control and curcumin treatment significantly reduced the expression of both parameters in CUR+DOX group (*p* < 0.05).

## 4. Discussion

Dox is an effective chemotherapeutic agent in many cancer types. However, its cardiotoxic effect limits its potential in cancer treatment. Due to the nature of the Dox-induced cardiotoxicity, certain antioxidant agents may be beneficial in alleviating its toxicity. Hence, the potential of curcumin, as an antioxidant agent, in Dox-induced cardiotoxicity was investigated in this study.

The findings of this study demonstrate that Dox-induced cardiotoxicity significantly increases the level of cTn. cTn is a well-accepted marker for myocardial injury, and hence commonly used in clinical cases. Increased cTn level can be seen in both acute and chronic heart failure and can even be correlated with high mortality in certain heart diseases [21]. Increased cTn level was also shown as a result of Dox use in many investigations [22,23]. In this study, curcumin use before Dox administration significantly reduced cTn level, which indicates a cardioprotective effect.

In this study, the electrocardiogram revealed a significant increase in QT interval in Dox-given rats. A prolonged QT interval is well associated with myocardial dysfunction and hence with cardiovascular-disease-related death [24]. The association of longer QT intervals with left ventricular systolic dysfunction was described previously [25]. We also observed increases in heart rate, systolic, and diastolic blood pressures in Dox-given rats as compared to the control. These findings indicate significant degenerative changes in the heart, most probably the development of acute myocardial failure with Dox administration. Left ventricular systolic dysfunction and eventually development of congestive heart failure is the most commonly described side effect of Dox [26]. In ECG, the effect of curcumin administration was only observed in the QT interval, which was stored to the level of the control group, indicating an improvement in the heart muscle’s ability to regain its function. Non-specific ST-T changes and decreased amplitude of QRS interval were also occasionally described in Dox toxicity [27]. However, no significant changes in QRS interval were observed in this study. Additionally, no significant changes were observed for heart rate, systolic, and diastolic blood pressure levels with curcumin administration. Although arrythmias have been reported in Dox-induced toxicity [28] no changes were detected in any of the groups studied in this investigation.

Dox-induced degenerative changes in heart histomorphology mostly include myocyte vacuolization, cell death, myofibrillary loss, matrix disorganization, and fibrosis. Depending on the dose and time, these changes show differences among the individuals. In experimental studies, myocyte vacuolization and death by necrosis and/or apoptosis are the most commonly reported findings in Dox-induced acute cardiomyopathies [2,29]. Similarly, vacuolated myocytes were abundant in Dox-given rats in this study and there were some myocytes with lost nuclei indicative of necrosis. Matrix disorganization was also evident in areas of degenerative cells. These findings clearly show myocyte degeneration in Dox use. Significant inflammatory changes and fibrosis, which were indicated in some studies [30,31], were not noted in our study. We also did not detect any changes in the levels of TNF-α, and IL-1β, which are key indicators of inflammatory response, in any of the groups in this study. However, there are plenty of studies indicating increased levels of these markers in Dox toxicities [32,33,34].

In dox-induced toxicities, cytokine elevation is a time-dependent phenomenon and mostly develops at later stages when inflammatory cells recruitment is pronounced. Cumulative dosing regimen in chronic toxicities are also associated with increased cytokine production [35,36]. However, in acute toxicities, oxidative stress and cell death are more significant [37]. Therefore, the findings of our study comply with acute toxicity development.

Hyperemia observed in the heart tissues of Dox-given rats in this study was not described previously. Although a significant inflammatory reaction was not observed in the Dox-given rats, the presence of hyperemia might be associated with early response. Curcumin administration significantly alleviated the degenerative changes observed in the Dox-given rats though some minor degenerative changes were still observable.

The exact mechanism of Dox-induced cardiac degeneration is still not fully understood. Dox as a chemotherapeutic agent works through inducing DNA damage, followed by blocking transcription and replication processes in cancer cells [38]. However, cardiotoxicity of Dox is rather induced by free radical production. Dox is reduced by NADH dehydrogenase in mitochondrial respiratory complex I and results in the formation of semiquinone radical which, can then react with molecular oxygen. Hence, the production of superoxide radical (O_2_^−^) takes place, and then through redox cycling, hydrogen peroxide (H_2_O_2_) and hydroxyl radical are produced. Additionally, through Fenton reaction, hydroxyl radical is produced from H_2_O_2_ [39]. Therefore, reactive oxygen species are the major constituents of the Dox-induced cellular degeneration. Cells or tissues, such as cardiomyocytes, with limited antioxidant capacities are more prone to Dox-induced degeneration [40].

Oxidative- and nitrosative-radicals-induced cellular degeneration is involved in almost all type of cellular insults. The level of the production of these radicals and the statue and capacity of the cellular defense mechanisms decide the fate of the cell, which may die or escape from death. Lipid peroxidation is one of the most important results of the reactive oxygen species activity. Lipid peroxidation, especially on cellular membranes, are not only responsible for histomorphological changes but also impair transmembrane signal transduction, hence the physiological function of the cell. MDA as an indicator of lipid peroxidation was shown to increase significantly in doxorubicin cardiotoxicity [12,31,41]. We also detected a significant increase in MDA level in the heart tissue in Dox-given rats. Curcumin use significantly reduced the MDA level similar to the level of the C group, indicating an alleviating effect on lipid peroxidation. GSH level and activities of SOD and CAT are important parameters to show antioxidant potential and statue of the tissues. GSH was indicated to be involved in Dox-induced toxicity [38]. A decreased level of GSH was reported in Dox cardiotoxicity [22,31] and curcumin administration was shown to increase the level of it [12]. Although a slight decrease in GSH level was observed in Dox-given rats, this change was not statistically significant, and no evident differences were detected among the groups in this study.

This study revealed significant decrease in CuZn-SOD and an increase in CAT activities in Dox-given rats. The decreased CuZn-SOD activity clearly indicates the depletion of the antioxidant defense mechanism, yet curcumin administration did not significantly improve the CuZn-SOD level in this study. The decreased CuZn-SOD activity in Dox cardiotoxicity was shown previously [4,22,41,42] though there are also studies reporting increased SOD activity with Dox use [12]. CAT activity was seen to increase with Dox use, and yet curcumin use was still not able reduce the CAT activity in this study. Increased CAT activity in Dox cardiotoxicity was also reported by others [42,43]. However, there are also many studies reporting decreased [22,41] and unchanged [44] CAT activity with Dox cardiotoxicity. Curcumin use was also previously reported to attenuate CAT activity in Dox toxicity [12,42]. Our findings on CAT activity therefore partially correlate with the previous studies.

The findings indicated that administration of Dox resulted in a significant increase in iNOS and nitrotyrosine immunoreactivities in rat heart tissue. The increased expression of iNOS is induced by cellular insults as a result of a defense mechanism. NOS is responsible for the catalytic formation of nitric oxide (NO) from L-arginine [45]. Increased production of O_2_^−^ and NO as a result of oxidative stress in cells causes peroxynitrite (ONOO^−^) formation. ONOO^−^ can oxidize lipoproteins and cause tyrosine nitration in many cellular proteins, causing degeneration in their structure and activity. Nitrotyrosine can indirectly detect the increased production of ONOO^−^ in tissues. Increased production of both iNOS and nitrotyrosine are clear indications of nitrosative tissue degeneration. Oxidative and nitrosative tissue degenerations occur mostly concomitantly as seen in this case and contribute significantly to the destruction of cellular structures. The increased level of NO and iNOS clearly indicates that Dox causes nitrosative tissue degeneration, and curcumin use alleviates their expression level, yielding some protective effect. Dox-induced increased production of NO and iNOS level was also shown by others [32,46]. Moreover, iNOS was reported to be necessary for the cytotoxic and immunogenic effects of Dox [47].

Many investigations have indicated the involvement of NF-κB in Dox-induced toxicity [32]. Moreover, it has been reported that NF-κB may even contribute to the resistance to Dox treatment in some cancer types [48]. As a nuclear transcription factor, NF-κB plays very important roles in many cellular processes [49]. Increased *NF-κB* expression in Dox cardiotoxicity was previously shown, and inhibition of *NF-κB* expression was reported to diminish Dox-induced apoptosis in adult rat cardiomyocytes [50]. It was also indicated that the p38 MAPK/NF-κB pathway is important in the induction of Dox-induced inflammation [51]. A significant increase in NF-κB mRNA expression was detected in rats treated with Dox compared to the control group, confirming its role in Dox-induced toxicity. It has also been detected that curcumin use before Dox significantly reduced the expression level of *NF-κB*, contributing to alleviating the degenerative effects caused by Dox.

Apelin and its receptor APJ are expressed highly in heart tissue [52]. It has been suggested that the Apelin–APJ signaling pathway may play an important role in cardiovascular homeostasis [53]. Being a powerful inotrope and effector on fluid homeostasis, Apelin’s role as a target for cardiovascular diseases bears importance. It has been shown that Apelin expression decreases heart failure [54]. This study also revealed a significant decrease in mRNA expression of *Apelin* in Dox-given rats. One of the main mechanisms of Dox-induced cellular degeneration takes place through the interruption of mitochondrial membrane integrity [55]. Dox-induced disruption of mitochondrial ion channels in cardiomyocytes is essentially important since transport of calcium required for muscle contraction fails. Apelin is reported to be involved in the management of calcium ions [56,57]. Therefore, stimulating the apelinergic system was suggested for therapeutic purposes in some cardiac diseases. Apart from decreased Apelin expression in Dox cardiotoxicity, this study also showed that curcumin use significantly increases the *Apelin* mRNA expression in Dox-given animals, promising a beneficial effect on correcting the muscle contraction.

The findings of this study confirm that curcumin has some antioxidant properties. Curcumin possesses several functional molecules including methoxy and phenoxy groups that provide its antioxidant property. It is denoted as a classical phenolic antioxidant since it donates H atom from the phenolic groups [58]. Antioxidant capacity of curcumin was shown in numerous studies. The increased expression level of several antioxidant enzymes such as SOD, CAT, and GPx with curcumin use in experimental studies has indicated that it strengthens the antioxidant defense system of cells [59]. Although the immunohistochemical analysis in the present study confirms that iNOS and nitrotyrosine levels, as a sign of oxidative and nitrosative stress, decreased in the CUR+DOX group, indicating a protective effect, no significant changes were observed in SOD and CAT levels. On the other hand, the significantly decreased MDA level with curcumin treatment clearly showed the antioxidant potential of curcumin.

## 5. Conclusions

Overall, the results of this study strongly suggest that curcumin may show alleviating effects in disrupted cardiac morphology, function, and the biochemical parameters that are indicative of oxidative stress that are induced by Dox treatment. We also showed that Apelin plays a significant role in Dox-induced cardiotoxicity and antioxidant agents that are effective on apelinergic system may be beneficial in the fight against Dox toxicity. Inhibition of the apelinergic pathway may be the further approach to investigate the effects of apelin in cardiotoxicity.

## Figures and Tables

**Table 1:** The primer sequences and the product sizes for *Actb*, *Apelin*, and *NF-кB*.

Genes	Primer Sequences.	Gene Ref. Seq. Number	Product Size (bp)
*Actb*	F: 5′-ATGAGCTGCCTGACGGTCAGGT-3′ R: 5′-GTGACGTTGACATCCGTAAAGACC-3′	NM_031144.3	139
*Apelin*	F: 5′-TGCCTCTTGCCTTATTAGCCTGC-3′ R: 5′-CTTCTGTTTCTATCTCTCCTCT-3′	NM_031612.3	93
*NF-кB*	F: 5′-TTCTCCGCCCGCGCCGCAGCCA-3′ R: 5′-GCTTCCGCGCCTGCGGGCTCCCG-3′	NM_001276711	103

F, forward primer; R, reverse primer.

**Table 2:** Electrocardiographic changes in the treatment groups.

Groups	HR (Beats/Min)	SBP (mm-Hg)	DBP (mm-Hg)	MBP (mm-Hg)	PR Interval (ms)	QRS Duration (ms)	QT Interval (ms)
C	312 (295–339) ^a^	76 (67–94) ^a^	72 (63–90) ^a^	74 (65–92) ^a^	30 (30–35) ^a^	51 (48–56) ^a^	88 (78–90) ^a^
CUR	319 (285–363) ^a^	89 (76–99) ^a^	85 (72–94) ^a^	87 (75–97) ^a^	34 (31–35) ^a^	46 (44–53) ^a^	86 (81–90) ^a^
DOX	400 (390–410) ^b^	117 (112–129) ^b^	111 (109–122) ^b^	115 (111–127) ^b^	31 (28–34) ^a^	54 (48–56) ^a^	97 (88–108) ^b^
CUR+DOX	400 (327–420) ^b^	115 (103–116) ^b^	108 (97–113) ^b^	111 (101–115) ^b^	32 (30–34) ^a^	48 (47–56) ^a^	86 (83–89) ^a^

Data are summarized as median (interquartile range; 25–75%). Letters that differ from each other in the same column are statistically significant (*p* < 0.05). C: control, CUR: curcumin, DOX: Doxorubicin, HR: heart rate, SBP: systolic blood pressure, DBP: diastolic blood pressure, MBP: mean blood pressure.

**Table 3:** Heart and serum tissue ELISA results.

Groups	Apelin (ng/L)		IL-1β (ng/L)		TNF-α (ng/L)		Troponin (ng/L)
	Serum	Heart	Serum	Heart	Serum	Heart	Serum
C	1794 (1556–1969) ^a^	4081 (4006–4219) ^bc^	26 (23–30) ^a^	51 (46–54) ^a^	128 (114–148) ^a^	197 (191–214) ^a^	79 (71–95) ^b^
CUR	1957 (1719–2075) ^a^	4194 (3969–4425) ^ac^	27 (18–41) ^a^	50 (48–53) ^a^	121 (115–136) ^a^	215 (212–226) ^a^	86 (63–103) ^b^
DOX	1357 (1094–1619) ^b^	3982 (3581–4106) ^b^	22 (19–25) ^a^	56 (50–60) ^a^	112 (85–145) ^a^	214 (201–226) ^a^	113 (92–106) ^a^
CUR+DOX	1931 (1681–2094) ^a^	4425 (4169–4794) ^a^	24 (20–25) ^a^	49 (47–52) ^a^	116 (91–138) ^a^	202 (192–209) ^a^	97 (93–101) ^ab^

Data are summarized as median (interquartile range; 25–75%). Letters that differ from each other in the same column are statistically significant (*p* < 0.05). C: control, CUR: curcumin, DOX: Doxorubicin, IL-1β: interleukin-1 beta, TNF-α: tumor necrosis factor alpha.

**Table 4:** Group comparison findings on biochemical analyses for the heart.

Groups	MDA (nmol/gwt)	GSH (nmol/gwt)	CuZn-SOD (U/g Protein)	CAT (K/g Protein)
C	172.4 (159.5–177.5) ^a^	979.5 (927.3–1113.6) ^a^	248.1 (186.4–287.7) ^a^	2.3 (2–2.8) ^a^
CUR	182.9 (178.3–188.8) ^a^	1038.6 (979.5–1063.6) ^a^	273.4 (244.9–294.2) ^a^	2.1 (1.9–2.3) ^a^
DOX	204.5 (175.9–211.1) ^b^	988.6 (922.7–1118.2) ^a^	210.8 (152.8–253.1) ^b^	3.0 (2.5–3.2) ^b^
CUR+DOX	171.1 (154.1–196.3) ^a^	954.5 (895.5–1036.4) ^a^	161.2 (137.8–183.1) ^b^	2.9 (2.5–3.1) ^b^

Data are summarized as median (interquartile range; 25–75%). Letters that differ from each other in the same column are statistically significant (*p* < 0.05). MDA: Malondialdehyde, GSH: Glutathione, CuZn-SOD: Copper Zinc Superoxide Dismutase, CAT: Catalase, C: Control, CUR: curcumin, DOX: Doxorubicin, gwt: gram wet tissue.

## Data Availability

The data that support the findings of this study are available from the corresponding authors upon reasonable request.

## Abbreviations

The following abbreviations are used in this manuscript:

Dox	Doxorubicin
MDA	Malondialdehyde
GSH	Glutathione
SOD	Superoxide dismutase
CAT	Catalase
GPx	Glutathione peroxidase
ROS	Reactive oxygen species
TNF-α	Tumor necrosis factor alpha
cTn	Troponin
NF-κB	Nuclear Factor Kappa B
ECG	Electrocardiography
iNOS	Inducible nitric oxide synthase
NO	Nitric oxide
